# Preparation of Antioxidant Peptides from Chicken Bone Proteins and the Influence of Their Compositional Characteristics on Antioxidant Activity

**DOI:** 10.3390/foods13244171

**Published:** 2024-12-23

**Authors:** Yitong Jin, Peng Zhou, Chengzhi Zhu, Yanan Liu, Zhijun Zhao

**Affiliations:** 1 College of Environmental Science and Engineering, Donghua University, 2999 North Renmin Road, Shanghai 201620, China; 2222256@mail.dhu.edu.cn; 2 Lab of Biorefinery, Shanghai Advanced Research Institute, Chinese Academy of Sciences, Shanghai 201210, China; 211310154@st.usst.edu.cn (P.Z.); 2021115041@nefu.edu.cn (C.Z.); 3 School of Health Science and Engineering, University of Shanghai for Science and Technology, Shanghai 200093, China; 4 School of Forestry, Northeast Forestry University, Harbin 150040, China

**Keywords:** chicken bone, enzymatic hydrolysis, amino acid composition, antioxidant peptides, molecular weights

## Abstract

Antioxidants play an important role in maintaining health and enhancing food stability by neutralizing free radicals. This study aimed to extract antioxidant peptides from white-feathered chicken bones through enzymatic hydrolysis, optimize the enzymatic hydrolysis conditions, and further investigate the relevance between the amino acid composition, molecular weight, and antioxidant activity of the resulting chicken bone hydrolysate. Alcalase was the most effective enzyme for hydrolyzing cooked chicken bones compared with papain, pepsin, and trypsin, yielding hydrolysates with the highest DH and ABTS radical scavenging activity. The enzymatic conditions were optimized using single-factor experiments and response surface methodology (RSM). The optimal conditions were a substrate concentration of 10%, an enzyme-substrate ratio of 502.75 U/g, a hydrolysis temperature of 48.48 °C, and a hydrolysis time of 1.13 h. Under these conditions, the ABTS radical scavenging activity reached 83.43%. Amino acid composition analysis revealed that peptides from chicken bones were rich in glycine, glutamic acid, alanine, proline, and aspartic acid, which were associated with antioxidant functions. Among these peptides, those with a molecular weight below 3 kDa exhibited the highest antioxidant effects through membrane filtration. In summary, chicken bone hydrolysate exhibits potent antioxidant activity, nominating them for potential application as natural antioxidants investible in novel functional foods and pharmaceuticals.

## 1. Introduction

Reactive oxygen species (ROS), including hydrogen peroxide (H_2_O_2_), superoxide anion (•O_2_^−^), hydroxyl radical (•OH), and singlet oxygen (^1^O_2_), are essential participants in various physiological activities in humans [1]. However, adverse exogenous factors, such as environmental pollution, ultraviolet radiation, and disease onset, can significantly increase ROS levels. This leads to oxidative damage to biomolecules, including lipid peroxidation, protein denaturation, and DNA damage, which has harmful effects on cells and tissues [2,3]. Oxidative stress results from an imbalance between ROS production and antioxidant defense in the body and is closely associated with aging and the onset of various diseases, such as cancer and disorders of the respiratory, cardiovascular, neurological, and digestive systems [4,5].

The intake of natural antioxidants through diet can effectively mitigate the harmful effects of ROS and restore the body’s antioxidant capacity [6]. Among those, antioxidant peptides derived from animal and plant sources have garnered widespread attention owing to their sustainability, affordability, and minimal side effects [7]. Antioxidant peptides from various sources have been investigated, including fish [8], livestock and poultry meat [9], dairy [10], egg whites [11], and cereals [12]. The antioxidant activities of these peptides have been attributed to the synergistic effects of various properties, including their abilities to scavenge free radicals, eliminate intracellular ROS, inhibit lipid peroxidation, and chelate transition metals [13].

Chicken bone accounts for approximately 25% of the total weight of the chicken. It is a major byproduct of broiler slaughtering and processing, making it a potential source of protein due to its high protein content [14]. Typically, chicken bones are used in animal feed or processed into bone powder, chicken bone extract, and other forms as food additives. Recent studies have shown that bioactive peptides extracted from chicken hydrolysates have various health benefits, such as promoting wound healing [15], antioxidation [14], and anti-aging [16] properties. Antioxidant peptides have been extracted from chicken blood corpuscle using enzymatic hydrolysis with papain and flavorzyme [17]. The study has shown that collagen peptides derived from chicken feet effectively promote wound healing, and biofilms have been developed for wound treatment based on these peptides [15]. Chicken bones have also been enzymatically hydrolyzed to prepare collagen peptides, with findings indicating that oral intake of these peptides can delay skin aging [16]. Therefore, extracting bioactive peptides from chicken bones holds promising potential for application.

Enzymatic hydrolysis is an effective method for preparing bioactive peptides, characterized by its mild and easily controlled conditions [18,19,20]. The selection of the enzyme is crucial for extracting bioactive peptides [21]. Alcalase, papain, pepsin, and trypsin are proteases commonly used in producing bioactive peptides in the food industry [2,22]. Alcalase has a broad range of enzymatic cleavage sites, with a particular specificity for hydrophobic amino acids such as Tyr, Phe, Leu, etc. [23,24,25]. Pepsin specifically cleaves aromatic amino acids, such as Phe, and hydrophobic amino acids [26]. Alcalase and papain were used to hydrolyze the distilled wasted grains of Chinese Baijiu, producing peptides with angiotensin-converting enzyme inhibitory action [27]. Oil palm kernel protein has been hydrolyzed enzymatically with pepsin and trypsin to produce antioxidant peptides [28]. Moreover, enzymatic hydrolysis conditions, such as the pH, temperature, substrate concentration, enzyme-to-substrate ratio, and hydrolysis time, are particularly crucial. These conditions directly affect the size, quantity, and amino acid composition of the peptides in the hydrolysate. These factors ultimately influence the bioactivity and functionality of the peptides. Response surface methodology (RSM) is a cost-effective approach for analyzing the relationship between multiple predictor variables, which has been widely applied in food research to optimize extraction processes [29]. The experimental results can be clearly interpreted through response surface plots [30,31,32,33].

Previous studies on antioxidant peptides derived from animal byproducts have primarily concentrated on characterizing their activities, with limited exploration of their compositional characteristics and molecular mechanisms. Moreover, chicken bones have been less studied till now. Therefore, we developed a technique for preparing bioactive peptides from chicken bone using enzymatic hydrolysis. Alcalase, papain, pepsin, and trypsin were individually used to hydrolyze the chicken bone to determine the optimal enzyme. Based on single-factor experiments, the preparation conditions of chicken bone hydrolysate were optimized using RSM. The antioxidant activity of the chicken bone hydrolysate was subsequently evaluated by measuring ABTS radical scavenging activity, hydroxyl radical scavenging capacity, metal chelating activity, and reducing power [14]. Furthermore, the effect of molecular weight on antioxidant activity was investigated using ultrafiltration. The results can aid in using chicken byproducts, potentially contributing to the development of the food, pharmaceutical, and environmental fields.

## 2. Materials and Methods

### 2.1. Materials

Fresh white-feathered chicken leg bones were obtained from Fujian Sunner Development Co., Ltd. (Nanping, China). Alcalase (500,000 U/g) was obtained from Shandong Longcotte Enzyme Preparation Co., Ltd. (Linyi, China). Papain (400,000 U/g) was purchased from Nanning Doing-higher Biotechnology Co., Ltd. (Nanning, China). Pepsin (30,000 U/g) and trypsin (250,000 U/g) were purchased from Macklin Biochemical Co., Ltd. (Shanghai, China). ABTS radical scavenging assay kits were purchased from Shanghai Yuanye Biotechnology Co., Ltd. (Shanghai, China), and the pre-stained low molecular weight marker was obtained from Sangon Biotech Co., Ltd. (Shanghai, China). Other analytical-grade reagents were acquired (analytical grade) from Sinopharm Chemical Reagent Co., Ltd. (Shanghai, China).

### 2.2. Preparation of Chicken Bone Powder

Fresh chicken leg bones were obtained from Fujian Sunner Development Co., Ltd. (Nanping, China) and brought to the laboratory for further processing. This was the bone in the middle part of a chicken leg, called the tibia. The bones were thoroughly cleaned after removing the surface meat and fascia, then drained, air-dried, and crushed using a bone grinder (ZM 300, Retsch, Haan, Germany). The chicken bone power was then sieved through a 100-mesh screen and stored at −20 °C for further use. A total of 100 g chicken bone powder was soaked in 10% hexane for 12 h to defat the sample (repeated thrice). Subsequently, the bone powder was washed with distilled water, and the pH was measured using a pH meter (8362sc, HACH, Loveland, OH, USA) until it became neutral. Then, a 0.5% ethylenediamine tetraacetic acid solution was added to soak for 36 h (the solution was replaced every 12 h) for decalcification, followed by another wash with distilled water to reach a neutral pH. After freeze drying (10N, SCIENTZ, Ningbo, China), raw chicken bone powder was obtained for subsequent use. The chicken bone was boiled in water for approximately 30 min, and the above pretreatment steps of chicken bone were repeated to obtain cooked chicken bone powder for spare. Analysis of the basic components of chicken bone power, such as its ash, fat, and total protein levels, was conducted by the Chinese National Standards GB5009.4-2016, GB5009.6-2016, and GB5009.5-2016, respectively [34]. Each sample was determined in triplicate.

### 2.3. Preparation of Chicken Bone Hydrolysate and Determination of the Appropriate Proteases

#### 2.3.1. Preparation of Chicken Bone Hydrolysate (CBH)

The chicken bone powder was dissolved in distilled water to achieve a final substrate concentration of 10%. Alcalase, papain, trypsin, and pepsin were added at a 1000 U/g protein enzyme-to-substrate ratio. Hydrolysis was performed under each enzyme’s optimal pH and temperature conditions for 30 min. The reaction was then terminated by heating the mixture in a water bath at 95 °C for 10 min, and the hydrolyzed product was centrifuged (4 °C, 10,000× *g*, 15 min). After collecting and freeze-drying the supernatant, chicken bone hydrolysate (CBH) was produced. The degree of hydrolysis (DH) and ABTS radical scavenging capacity of CBH prepared with different proteases were evaluated. The optimal hydrolysis conditions for each enzyme were as follows: alcalase (pH 9.5, 55 °C), papain (pH 7.5, 55 °C), trypsin (pH 8.0, 37 °C), and pepsin (pH 2.0, 37 °C).

#### 2.3.2. Determination of the Degree of Hydrolysis (DH)

CBH was determined for the degree of DH using the OPA method [4] with some modifications. The ortho-phthalaldehyde (OPA) reagent (200 mL) was prepared by dissolving 160 mg of OPA in 4 mL of ethanol. Then, 7620 mg of sodium tetraborate, 200 mg of sodium dodecyl sulfate (SDS), and 176 mg of dithiothreitol (DTT) were added, followed by the addition of distilled water to make up the volume to 200 mL. Detailed calculation steps can be found in Text S1 in the Supporting Information (SI). The DH was expressed using Equation (1):

(1) $$\mathrm{DH}\left(\%\right)=(h_{1}-h_{0})/h_{\mathrm{tot}}\times 100$$

where *h*_0_ represents the hydrolysis degree of the sample before hydrolysis, and *h*_1_ represents the hydrolysis degree of the sample after hydrolysis. The *h_tot_* for this experiment was 7.6.

#### 2.3.3. SDS-PAGE

SDS-PAGE of cooked chicken bone hydrolysates treated with different enzymes was conducted using a 15% separating gel and a 5% stacking gel. Briefly, the hydrolysates were diluted to a protein concentration of 3 mg/mL in a sample buffer and boiled at 100 °C for 5 min. After cooling, 15 μL of the solution was loaded into the gel. The staining agent was Coomassie Brilliant Blue R-250 in methanol. The electrophoresis was performed using a mini-vertical electrophoresis system (Mini-PROTEAN^®^ Tetra, Bio-Rad, Hercules, CA, USA) at 120 V for the stacking gel and 160 V for the separated gel for 2 h. The standard protein 10–180 kDa was used as the molecular weight marker [35].

### 2.4. Single-Factor Experiment

Alcalase was chosen for further research based on the conclusions of the determination of the appropriate protease experiments. The effects of individual factors on the ABTS radical scavenging activity of CBH were investigated to determine the preliminary ranges for hydrolysis conditions, including the substrate concentration, enzyme-substrate ratio, hydrolysis temperature, and hydrolysis time. The design of the single-factor experiments was presented in Table S1 of the SI. The chicken bone hydrolysate from the single-factor experiment, which resulted under optimum enzymatic conditions, was named CBH-1.

### 2.5. Response Surface Methodology Design

The Box–Behnken design was employed to optimize the extraction process of CBH. Based on the preliminary results (Section 2.4), enzyme-substrate ratio (200, 600, and 1000 U/g), hydrolysis temperature (40 °C, 55 °C, and 70 °C), and hydrolysis time (30, 60, and 90 min) were selected as the independent factors, while the ABTS radical scavenging rate was designated as the response value. Each factor was evaluated at three levels (−1, 0, and 1). Based on the value ranges of these three variables, 17 sets of experiments were designed using the Design-Expert 10 software. The experiments were conducted in a randomized condition and repeated three times. Details are provided in Table S2 of the SI.

### 2.6. Antioxidant Activity Determination

The ABTS radical scavenging activity was measured using an ABTS assay kit (Shanghai Yuanye Biotechnology Co., Ltd.) [36]. The hydroxyl radical scavenging assay was slightly modified based on the approach described in a previous study [34]. The reducing power was calculated as previously described [37]. The Fe^2+^ chelating ability was evaluated with minor modifications in accordance with a previous study [38] with minor modifications. All the details were shown in Texts S2–S5 in the SI.

### 2.7. Preparation and Separation of Chicken Bone Hydrolysate Under Optimal Enzymatic Hydrolysis Conditions

The CBH-2 obtained under the optimal conditions after RSM (enzyme-substrate ratio 502.75 U/g, hydrolysis temperature 48.48 °C, and hydrolysis time 1.13 h) was named CBH-2. The ABTS radical scavenging ability, hydroxyl radical scavenging ability, reducing power, and Fe^2+^ chelating ability of CBH-2 at different concentrations (2, 4, 6, 8, and 10 mg/mL) were evaluated using 10 mg/mL ascorbic acid (VC) as a control.

In addition, CBH-2 was separated stepwise using ultrafiltration tubes (Millipore, Darmstadt, Germany) with molecular weight cutoffs of 10, 5, and 3 kDa, yielding four fractions. These fractions were freeze-dried for further use, and their peptide yields (calculated as dry weight) were determined. Subsequently, each fraction’s ABTS radical scavenging, hydroxyl radical scavenging, reducing power, and Fe^2+^ chelating ability were measured at different concentrations (2, 4, 6, 8, and 10 mg/mL).

### 2.8. Determination of Amino Acid Composition and Molecular Weight Distribution of CBH-2

First, 100 mg of the freeze-dried material was added into a hydrolysis tube, followed by 8 mL of HCl (6 mol/L), N_2_ drying, and vacuum sealing. After hydrolyzing for 24 h at 110 °C, the sample was neutralized using 4.8 mL of NaOH (10 mol/L). The solution was later filtered through a 0.22 μm membrane after being diluted to 25 mL with deionized water. After that, 400 μL of the sample was extracted using an amino acid analyzer (MembraPure A300, Berlin, Germany) to examine the hydrolysis products. Owing to sample oxidation by formic acid before hydrolysis in HCl, tryptophan was broken down and was not detectable with this approach. Alcalase hydrolysis was performed to measure the tryptophan concentration, where 8 mL of 5 mol/L NaOH was used for hydrolysis, and 6.7 mL of 6 mol/L HCl was used for neutralization. Other steps were identical to the acid hydrolysis method. The final results of the amino acid composition results were expressed in mg/g of protein.

### 2.9. Statistical Analysis

We conducted all experiments in triplicate and expressed the results as mean values ± standard deviation (SD). SPSS 22.0 software was used for data analyses. The basic composition between raw and cooked chicken bone was compared using the Student’s *t*-test. To analyze differences in antioxidant activities among various fractions, one-way analysis of variance (ANOVA) followed by Duncan’s multiple range test was applied, with *p* < 0.05 indicating statistically significant. Design-Expert 11.0 was used to examine the RSM data.

## 3. Results and Discussion

### 3.1. Pretreatment of Chicken Bone and Determination of the Appropriate Proteases

Chicken bones are rich in protein, particularly collagen, making them an ideal raw material for enzymatic hydrolysis [18]. The basic composition of white-feathered chicken bones after pretreatment in this study is shown in Table 1. The ash, fat, and total protein contents of raw chicken bones are 40.71, 15.73, and 43.56 g/100 g, respectively, while those of cooked chicken bones are 47.30, 14.40, and 38.30 g/100 g, respectively. The collagen content in raw and cooked chicken bones is similar, with 10.78% and 8.97% accounting for 24.77% and 23.50% of their total protein content, respectively.

This study used alcalase, papain, pepsin, and trypsin to hydrolyze raw and cooked chicken bones from white-feathered chickens. The proteases were evaluated by measuring the DH of the hydrolysates and their ABTS radical scavenging activity.

**Figure 1:**
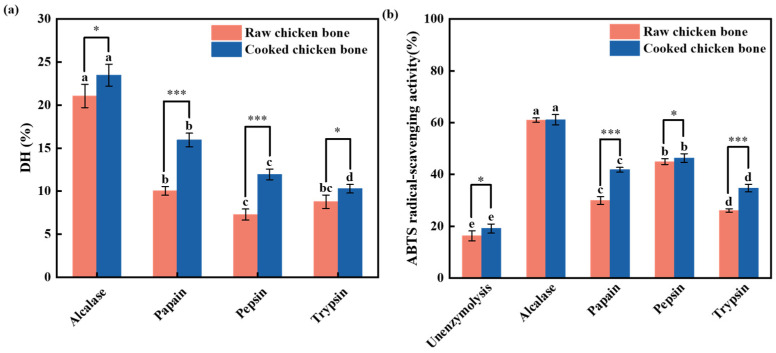
Degree of hydrolysis (DH) of chicken bone proteins (**a**) and ABTS radical scavenging activity of chicken bone hydrolysates treated with alcalase, papain, pepsin, and trypsin (**b**). * indicates *p* < 0.05 and *** indicates *p* < 0.001 between raw and cooked chicken bones within the same protease treatment. Different lowercase letters indicate significant differences (*p* < 0.05) among different protease treatments for the same chicken bone.

As shown in Figure 1a, the DH for CBH derived from hydrolyzing cooked chicken bones with alcalase, papain, pepsin, and trypsin is 23.49%, 15.95%, 11.94%, and 10.28%, respectively. Among these, the DH of the peptides hydrolyzed by alcalase is the highest, increasing by 32.09%, 49.15%, and 56.22% compared with the peptides hydrolyzed by the other three enzymes. A similar trend was observed for ABTS radical scavenging activity (Figure 1b). The ABTS radical scavenging activity of the peptides hydrolyzed by alcalase (61.10%) is 68.72%, 31.62%, 24.20%, and 43.20% higher than those of the untreated sample (19.11%), papain (41.78%), pepsin (46.32%), and trypsin (34.71%), respectively. This trend was also observed in raw chicken bones. Among hydrolysates derived from raw chicken bones, those obtained using alcalase exhibited higher DH and ABTS radical scavenging activity compared with those produced with papain, pepsin, or trypsin. The difference in DH and ABTS radical scavenging capacity between the cooked and raw chicken bones was most pronounced with papain and least significant with alcalase. Consequently, alcalase and cooked chicken bones were selected for further study.

**Figure 2:**
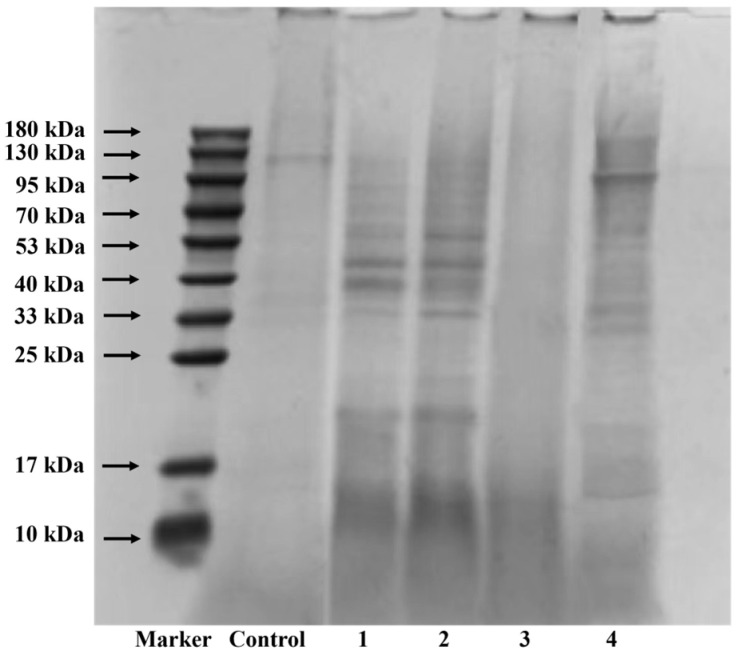
SDS-PAGE profiles of chicken bone proteins in the native form (lane Control) and after hydrolysis with pepsin (lane 1), trypsin (lane 2), alcalase (lane 3), and papain (lane 4) under optimal hydrolysis conditions for each enzyme.

Figure 2 illustrates the SDS-PAGE profiles of cooked chicken bone protein hydrolysates. The control group (Control) represents the total chicken bone proteins without enzyme addition. Lane Control showed one major band (130 kDa). The complete absence of protein bands (lane 3) in alcalase-prepared hydrolysates indicates that all degradation products are <10 kDa. The other three enzymes successfully degraded part of the chicken bone proteins, except for papain. The lowest molecular weight hydrolysate was observed in the alcalase hydrolysate, indicating a higher DH than the other proteases, which is consistent with the DH values. The antioxidant activity of peptides largely depends on the amino acid composition of their primary structure, particularly the presence of aromatic or hydrophobic amino acids and their residue content [39]. The alcalase used in this study is an endopeptidase derived from *Bacillus subtilis*, which preferentially hydrolyzes peptide bonds containing hydrophobic amino acids (e.g., Trp) and aromatic amino acids (Phe and Tyr) residues [24]. This mechanism helps delay lipid peroxidation by interacting with lipid or radical hydrophobic side chains [34]. Therefore, enzymatic hydrolysis of chicken bone using alcalase is conducive to generating more antioxidant peptides.

Alcalase and cooked chicken bones were selected as the enzyme and raw material for subsequent experiments.

### 3.2. Single-Factor Experiment

**Figure 3:**
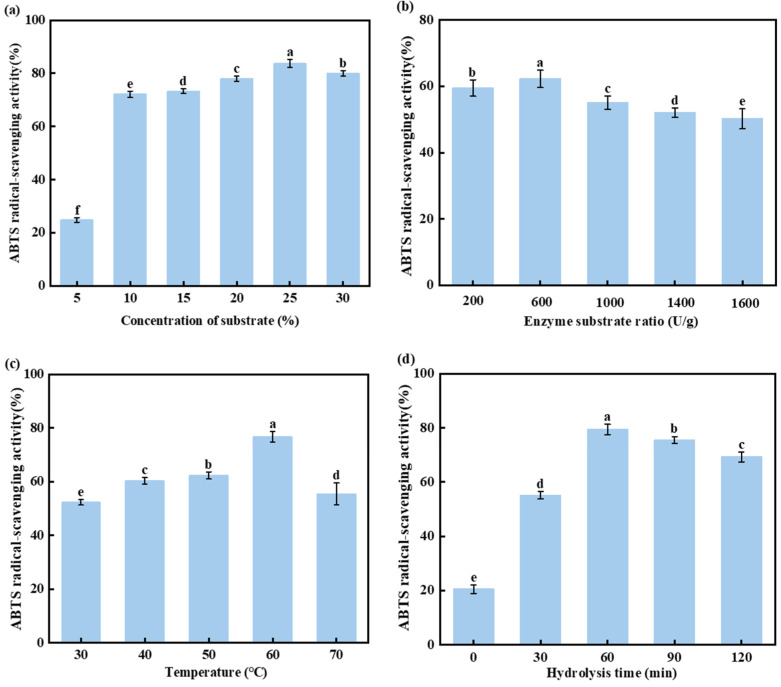
Effect of different substrate concentrations (**a**), enzyme-ratios (**b**), temperature (**c**), and hydrolysis time (**d**) on ABTS radical scavenging activity. Values without the same letter in each column have significant differences (*p* < 0.05).

The results of the single-factor experiments are shown in Figure 3, and the effects of substrate concentration, enzyme-substrate ratio, temperature, and time on the enzymatic hydrolysis reaction were investigated.

As shown in Figure 3a, the ABTS radical scavenging rate increased sharply as the substrate concentration increased from 5% to 10%. Subsequently, as the substrate concentration increased, the ABTS radical scavenging rate gradually increased, reaching a peak of 83.70% at a substrate concentration of 25%. However, within 120 min, the ABTS radical scavenging rate declined when the substrate concentration continued to increase. This could be due to insufficient enzyme availability to catalyze the substrate reaction. In addition, the high viscosity of the hydrolysate might impede the free diffusion of proteins and reduce enzymatic efficiency, resulting in a lower ABTS radical scavenging rate [40].

As shown in Figure 3b, the enzyme-substrate ratio significantly affects the antioxidant activity of the hydrolysates. At an enzyme-substrate ratio of 600 U/g, the ABTS radical scavenging rate reached its maximum (62.25%). Appropriately increasing the enzyme-substrate ratio can enhance the interactions between the substrate and the enzyme’s active sites. This allows more enzyme molecules to surround the substrate, facilitating the hydrolysis of large molecules into smaller peptides [41]. The ABTS radical scavenging rate decreased when the enzyme-substrate ratio exceeded 600 U/g. As the enzymatic reaction progressed, the substrates were gradually decomposed, leading to enzyme saturation and the reaction reaching its maximum rate. In addition, the limited substrate continues to be broken down into short peptides, which may also result in reduced antioxidant activity [42]. Furthermore, adding excessive enzymes may lead to resource wastage.

As shown in Figure 3c, the ABTS radical scavenging rate increases as the hydrolysis temperature increases from 30 °C to 60 °C, reaching its peak at 60 °C. However, the ABTS radical scavenging rate started to decline when the temperature exceeded 60 °C. This was because, within the normal temperature range, the rise in temperature increases the internal energy of enzyme molecules, promoting the efficiency of antioxidant peptide production. However, excessively high temperatures may alter the structure of the enzyme’s active site, preventing it from binding to the substrate and hindering the catalytic reaction [43], thereby causing a slight decrease in the ABTS radical scavenging rate.

Enzymatic hydrolysis time is an important factor influencing enzyme reactions (Figure 3d). Within the first 60 min, the ABTS radical scavenging rate increased rapidly with a prolonged hydrolysis time, reaching a maximum of 79.30% at 60 min. However, as the hydrolysis time continued, the ABTS radical scavenging rate declined. This is because the hydrolysis time affects the mass transfer rate, which affects the hydrolysis efficiency [44]. In the early stages of hydrolysis, the release of soluble peptides in the hydrolysate is time- dependent. However, when the hydrolysis time exceeds 60 min, the saturation effect of the solvent on soluble peptides and prolonged hydrolysis may cause the highly active peptide fragments generated initially to be further degraded into smaller peptides or free amino acids, thereby reducing the antioxidant activity of the hydrolysate [45].

Therefore, based on the single-factor experimental results, the optimal hydrolysis conditions were selected as follows: a substrate concentration of 25%, an enzyme-substrate ratio of 600 U/g, a temperature of 60 °C, and a hydrolysis time of 60 min. Under these conditions, the ABTS radical scavenging rate of chicken bone hydrolysate (CBH-1) was 62.30%.

### 3.3. Optimization of the Hydrolysis Parameters Using RSM

The results of the single-factor experiments indicated that substrate concentration, enzyme-substrate ratio, hydrolysis temperature, and hydrolysis time were key factors influencing the antioxidant activity of CBH. The experiment revealed that, compared with the other three factors, substrate concentration had a minimal effect on ABTS radical scavenging activity once it exceeded 10%. Therefore, the substrate concentration was set at 10%, and three significant factors (enzyme-substrate ratio, hydrolysis temperature, and hydrolysis time) were selected as independent variables, with ABTS radical scavenging activity as the response variable. Hydrolysis conditions were optimized using response surface methodology (RSM).

A regression analysis was performed on the experimental data with results summarized in Table 2. The regression equation for the ABTS radical scavenging rate (Y) of the hydrolysates about the influencing factors was obtained with Equation (2):

(2) $$\begin{array}{c} Y=78.6917-0.98240X_{1}-7.29388X_{2}+4.22518X_{3}+0.508247X_{1}X_{2}+0.989945X_{1}X_{3}\\ -\;1.91442X_{2}X_{3}-2.98891X_{1}^{2}-13.9409X_{2}^{2}{-5.1675X}_{3}^{2} \end{array}$$

**Figure 4:**
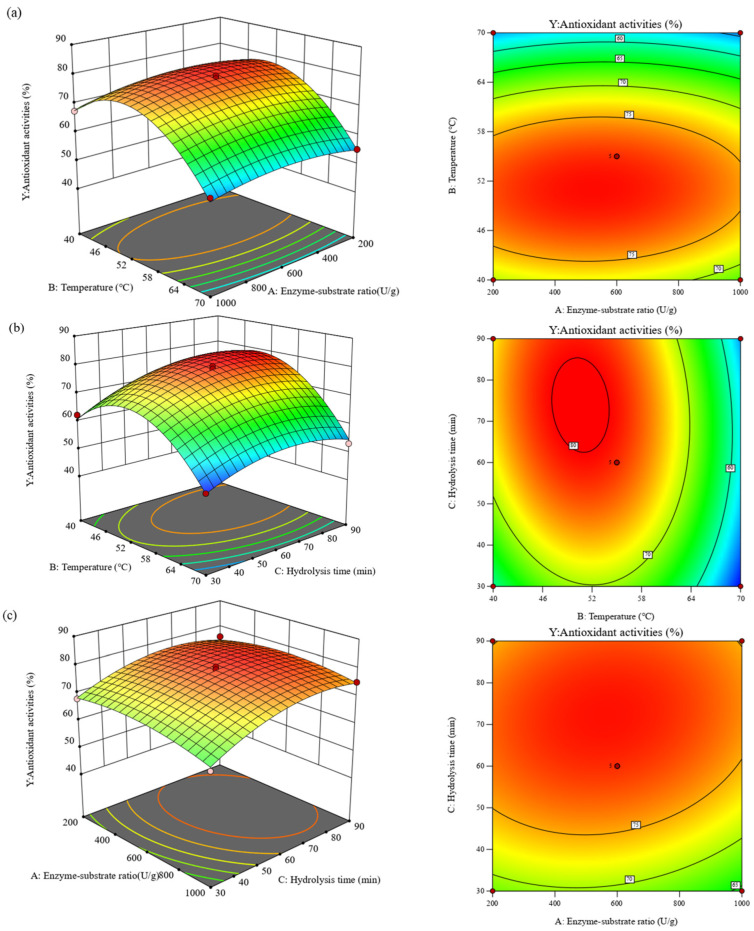
Response surface plots showing a correlation between (**a**) enzyme-substrate ratio and hydrolysis temperature, (**b**) hydrolysis temperature and hydrolysis time, and (**c**) enzyme-substrate ratio and hydrolysis time on ABTS radical scavenging of CBH.

The ANOVA results of the fitted quadratic polynomial regression are presented in Table 3. The quadratic regression model was significant (*p* < 0.05), confirming its reliability for determining the ABTS radical scavenging activity. The lack of fit was insignificant (*p* = 0.1647 > 0.05), further supporting the validity of the model. Additionally, the correlation coefficient (R^2^) was 0.9900, suggesting a good accuracy of the model. The adjusted determination coefficient value (R^2^_adj_) was 0.9771, which also confirmed that the experimental values agreed with the predicted ones. Meanwhile, a relatively low value of the coefficient of variation (CV = 2.25) indicated a high degree of precision and reliability of the experimental results [44]. The model terms with smaller *p*-value (*p* < 0.05) and larger *F*-value had a greater impact on the extraction rate. Based on the *p*-value, the factors influencing the ABTS scavenging activity followed the order: X_2_ > X_3_ > X_1_. Among the variables, the two independent factors (X_2_ and X_3_), three quadratic terms (X_1_^2^, X_2_^2^, and X_3_^2^), and one interaction term X_2_X_3_ were significant (*p* < 0.05), whereas the other terms were not significant (*p* > 0.05). These findings suggest that the effects of enzyme-substrate ratio, hydrolysis temperature, and hydrolysis time on the ABTS radical scavenging activity of CBH were complex, with strong interaction effects. As illustrated in Figure 4, a significant interaction was observed between enzymatic temperature and time (Figure 4b). The steep slope of the three-dimensional response surface plots confirmed that enzymatic temperature had the most substantial effect on the ABTS radical scavenging rate, followed by enzymatic time, with the enzyme-substrate ratio exerting the least effect. These results aligned with the analysis of variance for the RSM.

The optimal process conditions obtained from the Box–Behnken model were an enzyme-substrate ratio of 502.75 U/g, an enzymatic temperature of 48.48 °C, and an enzymatic time of 1.13 h, yielding a theoretical ABTS radical scavenging rate of 80.25%. Validation under these conditions resulted in an actual ABTS radical scavenging rate of 83.43%, with a relative error of 3.81%, which was in close agreement with the predicted value. This consistency between experimental and predicted results affirms the model’s reliability and demonstrates that these conditions are conducive to improving the antioxidant properties of CBH.

### 3.4. Antioxidant Properties of Chicken Bone Hydrolysate Under Optimal Enzymatic Conditions

**Figure 5:**
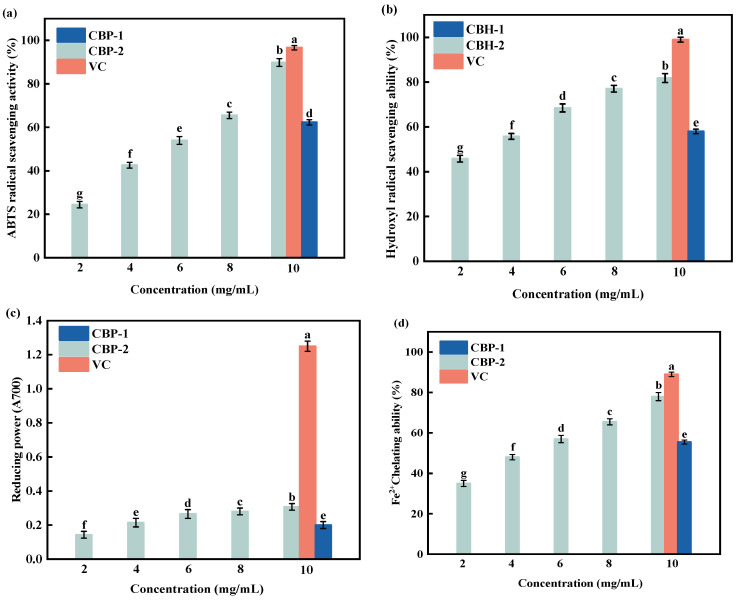
ABTS radical scavenging activity (**a**), hydroxyl radical scavenging activity (**b**), reducing power (**c**), and Fe^2+^ chelating capacity (**d**) of CBH at different concentrations were determined separately, with ascorbic acid (VC) at a concentration of 10 mg/mL used as a positive control. CBH obtained under the optimal single-factor experiment was named CBH-1, while the one optimized using RSM was named CBH-2. The results were expressed as the mean ± standard deviation of three determinations. Values with different letters differ significantly (*p* < 0.05).

The experimental results of the antioxidant activity of CBH-2 are shown in Figure 5, including the ABTS radical scavenging rate, hydroxyl radical scavenging rate, reducing power, and Fe^2+^ chelating capacity. All four antioxidant capacities exhibited a dose-dependent increase. As the concentration of CBH-2 increased from 2 to 10 mg/mL, the ABTS radical scavenging rate increased from 24.43% to 89.84%. At 10 mg/mL, the ABTS radical scavenging rate of chicken bone hydrolysate (89.84%) was 44.21% higher than that of chicken bone hydrolysate (CBH-1) before response surface optimization (62.30%). Similarly, CBH-2 showed increases of 29.11% in hydroxyl radical scavenging rate (81.81%), 34.85% in reducing power (0.307), and 28.85% in Fe^2+^ chelating capacity (78.00%) compared with the preoptimized version. These results indicate that CBH-2 have good antioxidant properties. Moreover, CBH-2 exhibited a hydroxyl radical scavenging rate of 34.56% higher than that of camel peptides obtained under optimal enzymatic conditions (10 mg/mL) [15]. It also showed 22.10% and 40.06% higher ABTS radical scavenging rates than those from enzymatic hydrolysates of casein using alcalase (CNH) and buffalo casein (CBH), respectively [24]. In addition, CBH-2 had a 16.57% higher superoxide anion radical scavenging rate than bovine bone peptides obtained through ultrasound-assisted double protease hydrolysis (5 mg/mL). These findings further confirm the excellent antioxidant activity of CBH.

### 3.5. Characterization of CBH-2

#### 3.5.1. Amino Acid Composition of CBH-2

**Figure 6:**
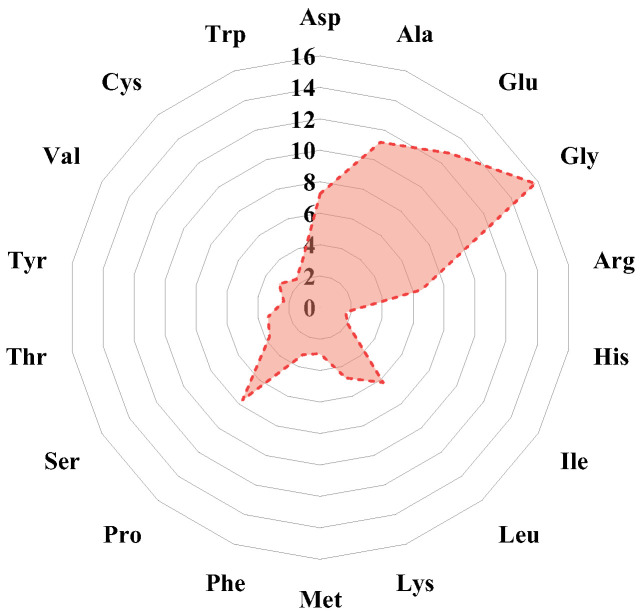
Amino acid composition of CBH-2.

Figure 6 illustrates the amino acid composition of CBH-2. CBH-2 contains significant quantities of glycine (82.67 mg/g), glutamic acid (67.13 mg/g), alanine (58.62 mg/g), proline (40.18 mg/g), and aspartic acid (37.95 mg/g), which aligns with previously reported finding [16]. This study demonstrates that the proportion of antioxidant amino acids can reach as high as 77.62%.

The antioxidant activity of peptides is generally influenced by their amino acid composition, especially those with antioxidant properties such as aromatic and hydrophobic amino acids (Glu, Ile, Ala, Val, Phe, Asp, Tyr, Pro, Trp, Gly, Met, and Leu) [46]. Glycine (Gly), characterized by a side chain containing only one hydrogen atom, plays a crucial role in the antioxidant activity of peptides [21]. Peptides containing hydrophobic amino acids enhance free radical scavenging activity by strengthening their interaction with lipids or acting as proton or hydrogen donors. For instance, alanine promotes the solubility of peptides in lipid matrices, facilitating interaction with radicals in the lipid phase [21], while the pyrrolidine ring in proline (Pro) can act as a hydrogen donor to scavenge free radicals. Moreover, glutamic acid (Glu) and aspartic acid (Asp) have extra electrons that can quench unpaired electrons or free radicals, thereby contributing to their antioxidant effects [47]. Therefore, the high antioxidant activity of CBH may be closely related to their amino acid composition.

In addition, amino acid residues of peptides such as Gly, Pro, Asp, and Ala can interact with free radicals like DPPH and ABTS through hydrogen bonding and hydrophobic interactions to scavenge free radicals. For example, in the peptide SGPPVPGPIGPM from tilapia, Gly-7 was found to form hydrophobic interactions with DPPH, as well as a hydrogen bond with a length of 2.72 Å involving Val-5, Pro-6, Pro-8, and Ile-9 [48]. Similarly, the peptide HADMVFY from walnuts formed a 2.89 Å hydrogen bond with ABTS and interacted hydrophobically with the Ala-2 residue [49]. In watermelon seed peptide RDPEER, Arg-1 and Glu-5 residues formed two hydrogen bonds with ABTS, while Asp-2, Arg-6, and Glu-4 contributed to three types of hydrophobic interactions with the same molecule [50].

#### 3.5.2. Molecular Weight Distribution and Antioxidant Activity of CBH-2 with Different Molecular Weights

**Figure 7:**
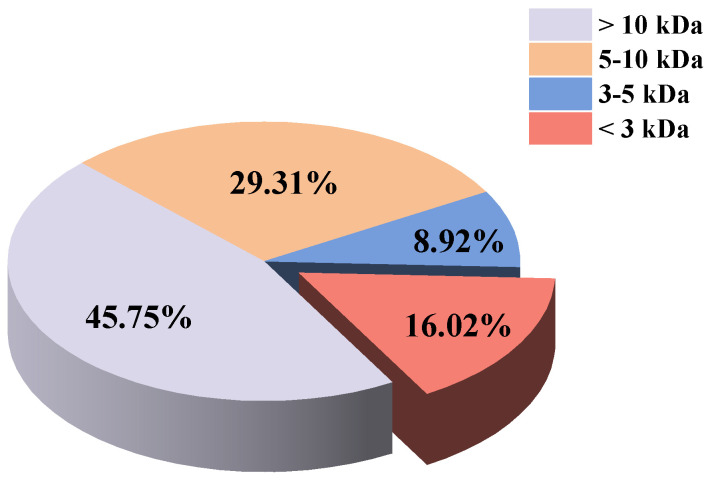
Molecular weight distribution of peptides of CBH-2.

**Figure 8:**
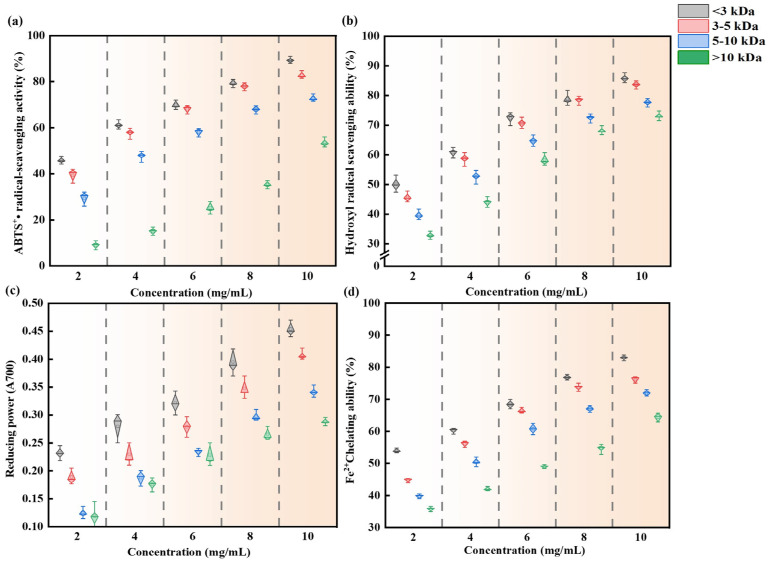
(**a**) ABTS radical scavenging, (**b**) hydroxyl radical scavenging, (**c**) reducing power, and (**d**) Fe^2+^ chelating capacity of CBH-2 of different concentrations and molecular weights.

The analysis of the molecular weight distribution of CBH-2 is depicted in Figure 7. Peptides with a molecular weight < 10 kDa accounted for 54.25%, those <5 kDa constituted 24.94%, and those <3 kDa comprised 16.02%. Previous studies have indicated that duck plasma protein peptides with a molecular weight < 3 kDa exhibit the highest antioxidant activity [5]. Similarly, higher antioxidant activity was observed in the lower molecular weight peptide fractions of Edible Bird’s Nest hydrolysate [51], soybean hydrolysate [38], and Jiuzao protein hydrolysates [52]. Bioactive peptides typically consist of 2 to 20 amino acid residues per molecule, and the lower their molecular weight, the more easily they exert biological effects [53]. Smaller peptides tend to have higher mobility and diffusibility, enabling them to interact more effectively with target molecules [54]. The results of this study confirm that peptides with a molecular weight < 3 kDa exhibit the highest ABTS radical scavenging rate, hydroxyl radical scavenging rate, reducing power, and Fe^2+^ chelating capacity, which were 89.26%, 85.88%, 0.45, and 85.96% (at 10 mg/mL), respectively (Figure 8).

In this study, antioxidant peptides were extracted from chicken bone hydrolysate, and the enzymatic hydrolysis conditions were optimized. We explored the relationship between the composition of the chicken bone hydrolysate and its antioxidant activity. However, the molecular mechanisms by which the chicken bone hydrolysates exert their antioxidant activity remain unclear. Future research is required to investigate the structure-function relationship of CBH-2 and use molecular docking to analyze the dynamic interactions between the peptides and target proteins, thus revealing their potential antioxidant mechanisms.

## 4. Conclusions

In summary, peptides with significant antioxidant activity were obtained from the cooked chicken bones of white-feather chicken through enzymatic hydrolysis with alcalase. The enzymatic hydrolysis conditions were optimized, resulting in significantly enhanced ABTS radical scavenging activity of CBH-2 (83.43%) compared with CBH-1 (62.30%). The presence of Gly, Glu, Ala, Pro, and Asp residues in the composition of CBH-2 could explain its higher antioxidant activities. These residues may bind to free radicals through hydrogen bonds and hydrophobic interactions, thereby exerting antioxidant effects. Peptides with molecular weights below 3000 Da, obtained through ultrafiltration, exhibited the strongest antioxidant properties. This study highlights the enhanced use of chicken bones for producing antioxidant peptides through optimized enzymatic hydrolysis. Extracting bioactive peptides from chicken bones contributes to the high-value utilization of chicken byproducts. Future research might focus on the structural elucidation and mechanism of action of these antioxidant peptides and broaden their application in value-added products.

## Figures and Tables

**Table 1:** The composition of raw chicken bone and cooked chicken bone ^a^.

Composition (g/100 g Dry Biomass)	Raw Chicken Bone	Cooked Chicken Bone
Ash	40.71 ± 0.20 ^b^	47.30 ± 0.10 ^a^
Fat	15.73 ± 0.10 ^a^	14.40 ± 0.10 ^b^
Total protein	43.56 ± 0.20 ^a^	38.30 ± 0.20 ^b^
Collagen	10.78 ± 0.10 ^a^	8.97 ± 0.10 ^b^

Note: All data were expressed as mean ± SD (n = 3); different letters within the same column indicate significant differences between raw and cooked chicken bone (*p* < 0.05).

**Table 2:** Response surface design and experimental results.

Group	Code Level of Variables			Response Value (Y)
	Enzyme-Substrate Ratio (U/g)	Temperature (°C)	Hydrolysis Time (min)	ABTS Radical Scavenging Activity (%)
1	1	0	−1	68.01
2	0	1	1	53.11
3	1	0	−1	63.01
4	0	−1	1	72.91
5	0	0	0	79.33
6	0	1	1	50.11
7	1	−1	0	67.40
8	0	0	0	79.01
9	−1	0	1	76.10
10	1	0	1	75.07
11	0	−1	−1	62.25
12	1	1	0	55.21
13	−1	1	0	55.11
14	0	0	0	78.01
15	−1	−1	0	69.33
16	0	0	0	77.11
17	0	0	0	80.01

**Table 3:** Analyze of variance for the second-order polynomial model.

Source	Sum of Squares	df	Mean Square	F-Value	*p*-Value	Significance
Model	1630.03	9	181.11	76.86	<0.0001	significant
X_1_	7.72	1	7.72	3.28	0.1132	NS
X_2_	425.60	1	425.60	180.61	<0.0001	***
X_3_	142.82	1	142.82	60.61	0.0001	***
X_1_X_2_	1.03	1	1.03	0.4385	0.5290	
X_1_X_3_	3.92	1	3.92	1.66	0.2381	
X_2_X_3_	14.66	1	14.66	6.22	0.0413	*
X_1_^2^	37.61	1	37.61	15.96	0.0052	**
X_2_^2^	818.31	1	818.31	347.27	<0.0001	***
X_3_^2^	111.97	1	111.97	47.52	0.0002	***
Residual	16.49	7	2.36			
Lack of fit	11.31	3	3.77	2.91	0.1647	NS
Pure error	5.19	4	1.30			
Cor total	1646.53	16				
R^2^	0.9900					
R^2^_Adj_	0.9771					
CV/%	2.25					

Note: X_1_, X_2_, and X_3_ are enzyme-substrate ratio, temperature, and hydrolysis time, respectively. * *p* < 0.05, ** *p* <0.01, *** *p* < 0.001, NS: not significant.

## Data Availability

The original contributions presented in this study are included in the article/Supplementary Materials; further inquiries can be directed to the corresponding authors.

## Supplementary Materials

The following supporting information can be downloaded at: https://www.mdpi.com/article/10.3390/foods13244171/s1. Supplementary data associated with this article can be found in the supporting information. Text S1: Determination of the degree of hydrolysis (DH); Text S2: ABTS radical scavenging assay; Text S3: Hydroxyl radical scavenging assay; Text S4: Determination of reducing power; Text S5: Fe^2+^ chelating ability assay; Table S1: Single-factor test conditions; Table S2 Box–Behnken test factors and level.
