# Supplementary material for: Preparation of Antioxidant Peptides from Chicken Bone Proteins and the Influence of Their Compositional Characteristics on Antioxidant Activity
Source: Foods. 2024 Dec 23;13(24):4171. doi: 10.3390/foods13244171 (PMC11675203; doi:10.3390/foods13244171)
Supplement: Supplementary file 1 [file foods-13-04171-s001.zip › foods-3357670-supplementary.pdf]

# Supporting Information

## Preparation of antioxidant peptides from chicken bone proteins and the influence of their compositional characteristics on antioxidant activity

Yitong Jin[1,2], Peng Zhou[2,3], Chengzhi Zhu[2,4], Yanan Liu[1,*], Zhijun Zhao[2,*]

*([1.] College of Environmental Science and Engineering, Donghua University, 2999 North Renmin Road, Shanghai 201620, China.*

*[2.] Lab of Biorefinery, Shanghai Advanced Research Institute, Chinese Academy of Sciences, Shanghai 201210, China.*

*[3.] School of Health Science and Engineering, University of Shanghai for Science and Technology, Shanghai 200093, China.*

*[4.] School of Forestry, Northeast Forestry University, Harbin 150040, China)*

*Corresponding author: Tel: +86 21 67792538; Fax: +86 21 67792522;

E-mail: liuyanan@dhu.edu.cn (Yanan Liu), zhaozj@sari.ac.cn (Zhijun Zhao)

**9 Pages**

**5 Texts (S1-S5)**

**2 Tables (S1–S2)**

## Text

### Text S1 Determination of the degree of hydrolysis (DH)

3 mL of OPA reagent was added to each test tube, 400 μL of serine standard solution was added to the test tube, and the absorption value $OD_{stand}$ was measured at 340 nm after 2 min. It was calculated according to Eqs. (1), (2), and (3):

$$SerineNH_2 = \left[\left(OD_{sample} - OD_{blank}\right) \times 0.9516 \times X\right] / \left[\left(OD_{sample} - OD_{blank}\right) \times Y \times P\right] \quad (1)$$

$$h = (SerineNH_2 - \beta)/\alpha \quad (2)$$

$$DH(\%) = (h_1 - h_0)/h_{tot} \times 100 \quad (3)$$

Where, $OD_{blank}$ represents the blank assay value, obtained by replacing the serine standard solution with deionized water, $OD_{sample}$ is the assay value when the serine standard solution is substituted with the sample, $OD_{stand}$ is the assay value of the serine standard solution, $X$: volume of the sample (L), $Y$: mass of the sample (g), $P$: protein content in the sample (%), $h_0$: hydrolysis degree of the sample before hydrolysis, $h_1$: hydrolysis degree of the sample after hydrolysis. The $\alpha$, $\beta$ and $h_{tot}$ for this experiment were 1.00, 0.40 and 7.6 respectively.

**Text S2 ABTS radical scavenging assay**

The ABTS radical scavenging activity was measured using an ABTS assay kit (Shanghai Yuanye Biotechnology Co., Ltd.) [1]. The working solution consisted of equal volumes of ABTS and oxidant solutions stored in the dark place at 25°C for 16 h. The solution was then diluted with deionized water until its absorbance at 405 nm reached 1.4 ± 0.05. Subsequently, 7 μL of the sample was added to the working solution, and the mixture was incubated at 25°C for 2–6 min. Deionized water (7 μL) was used as a blank. The absorbance of the mixtures was measured at 405 nm and used to calculate the total antioxidant capacity of the sample. The scavenging rate (%) was calculated using Eq. (4):

$$\textit{ABTS radical scavenging activity}\ (\%) = [(A_{blank} - A_s)/A_{black}] \times 100 \qquad (4)$$

where $A_{blank}$ was the absorbance of the blank at 405 nm and $A_s$ was the absorbance of the sample at 405 nm.

**Text S3 Hydroxyl radical scavenging assay**

The method was slightly modified based on the approach described in a previous study [2]. The specific steps are as follows: 1 mL of the sample (2–10 mg/mL) was mixed with 1 mL of salicylic acid solution (9 mmol/L), 1 mL of $FeSO_4$ solution (9 mmol/L), and 1 mL of $H_2O_2$ solution (8.8 mmol/L) and thoroughly mixed. The mixture was incubated at 37°C for 30 min. After cooling, the absorbance was measured at 510 nm. The control group used deionized water instead of the sample, while the blank group used deionized water instead of a salicylic acid solution. The hydroxyl radical scavenging rate was expressed as Eq. (5):

$$\text{Hydroxyl radical scavenging activity } (\%) = [(A_c + A_b - A_s)/A_c] \times 100 \quad (5)$$

where $A_s$ is the absorbance of the sample in the reaction, $A_b$ is the absorbance of water instead of the salicylic acid used, and $A_c$ is the absorbance of water instead of the sample.

**Text S4 Determination of reducing power**

The reducing power was determined using the slightly modified method [3]. For this, 1.0 mL of the sample (2–10 mg/mL) was mixed with 1.0 mL of phosphate buffer (0.2 M, pH 6.6) and 1.0 mL of 1% (w/v) potassium ferricyanide. After incubation at 50°C for 20 min, 1.0 mL of 10% (w/v) trichloroacetic acid was added to the mixture. The solution was then centrifuged, and 2.0 mL of the supernatant was mixed with 2.0 mL of distilled water and 0.8 mL of 0.1% (w/v) $FeCl_3$ and incubated for 10 min. The absorbance of the resulting solution was measured at 700 nm. An increase in the absorbance of the reaction mixture indicates an increase in the reducing power.

**Text S5 $Fe^{2+}$ chelating ability assay**

The metal ion-chelating activity was evaluated according to a previous study [4] with minor modifications. 0.5 mL of the sample (2–10 mg/mL) was mixed with 2 mL of $FeCl_2$ (2 mmol/L) and 1.6 mL of distilled water. After incubating for 30 s, 0.1 mL (5 mmol/L) of $FeCl_2$ was added, and the reaction was conducted at 25°C for 10 min. The absorbance was measured spectrophotometrically at 562 nm. Deionized water, with no sample added, was used for the blank. The result was expressed using Eq. (6):

$$Fe^{2+}\ chelating\ activity\ (\%) = [(A_0 - A_1)/A_0] \times 100 \quad (6)$$

where $A_0$ and $A_1$ represent the absorbance of the blank and the sample at 562 nm, respectively.

**Table**

**Table S1 Single factor test conditions**

| Group | Substrate concentration (%) | Enzyme-substrate ratio (U/g) | Hydrolysis time (min) | Hydrolysis temperature (°C) |
|---|---|---|---|---|
| 1 | 5%<br>10%<br>15%<br>20%<br>25%<br>30% | 1000 | 30 | 40 |
| 2 | 10% | 1000 | 30<br>60<br>90<br>120 | 40 |
| 3 | 10% | 1000 | 30 | 30<br>40<br>50<br>60<br>70 |
| 4 | 10% | 200<br>600<br>1000<br>1400 | 30 | 40 |

**Table S2 Box-Behnken test factors and level**

| Factors | Code | Level | | |
|---|---|---|---|---|
| | | -1 | 0 | 1 |
| Enzyme-substrate ratio (U/g) | $X_1$ | 200 | 600 | 1000 |
| Hydrolysis temperature (℃) | $X_2$ | 40 | 55 | 70 |
| Hydrolysis time (min) | $X_3$ | 30 | 60 | 90 |

## References

1. Zhang, J. H.; Qi, X.; Shen, M. Y.; Yu, Q.; Chen, Y.; Xie, J. H. Antioxidant stability and in vitro digestion of β-carotene-loaded oil-in-water emulsions stabilized by whey protein isolate-Mesona chinensis polysaccharide conjugates. *Food Res. Int.* **2023**, *174,* 113584.

2. Wang, J. N.; Yang, G.; Li, H. F.; Zhang, T.; Sun, D.; Peng Lu, W. P.; Zhang, W. J.; Wang, Y. H.; Ma, M.; Cao, X. F.; Zhang, B.; Guo, Y. C. Preparation and identification of novel antioxidant peptides from camel bone protein. *Food Chem*. **2023**, *424*, 136253.

3. Moon, S. H.; Cho, S. J. Evaluation of the antioxidant activity of Tetraselmis chuii after in vitro gastrointestinal digestion and investigation of its antioxidant peptides. *Algal Res*. **2023**, *76*,103328.

4. Zhang, Q. Z.; Tong, X. H.; Sui, X. N.; Wang, Z. J.; Qi, B. K.; Li, Y.; Jiang, L. Z. Antioxidant activity and protective effects of Alcalase-hydrolyzed soybean hydrolysate in human intestinal epithelial Caco-2 cells. *Food Res. Int*. **2018**, *111*, 256-264.
